# Advanced glycation end products impair the functions of saphenous vein but not thoracic artery smooth muscle cells through RAGE/MAPK signalling pathway in diabetes

**DOI:** 10.1111/jcmm.12886

**Published:** 2016-06-14

**Authors:** Yongxin Sun, Le Kang, Jun Li, Huan Liu, Yulin Wang, Chunsheng Wang, Yunzeng Zou

**Affiliations:** ^1^ Department of Cardiac SurgeryZhongshan HospitalFudan UniversityShanghaiChina; ^2^ Shanghai Institute of Cardiovascular DiseasesZhongshan HospitalFudan UniversityShanghaiChina

**Keywords:** advanced glycation end products, RAGE, mitogen activated protein kinase signalling pathway, smooth muscle cells, saphenous vein

## Abstract

Saphenous vein (SV) and internal thoracic artery (ITA) are commonly used bypass conduits. However, graft failure occurs in SV rather than in ITA, especially in diabetes (DM). The mechanism for this difference has not been fully understood. Accumulation of advanced glycation end products (AGEs) and activation of AGEs receptor (RAGE) could accelerate smooth muscle cells (SMC) proliferation in DM, we thus asked whether AGEs‐RAGE could mediate the differences between SMC from SV (SMC_V_) and from ITA (SMC_A_). Twenty‐five patients with DM and other 25 patients without DM were enclosed in DM and control group, respectively. AGEs (100 μg/ml) were added to cultured SMC_A_ and SMC_V_ obtained at coronary artery bypass graft (CABG) and proliferative rates were determined. Transcript expression, phosphorylation or protein expression levels of MAP kinase family (ERK, p38 and JNK), matrix metalloproteinases (MMP)‐2 and MMP‐9 were analysed by real‐time PCR, Western‐blot or immunofluorescence staining, respectively. Compared with paired SMC_A_, SMC_V_ showed significantly increased proliferation rate, MAP kinase family phosphorylation, and MMP‐2/9 expression in both groups, especially in DM group. The responses of SMC_V_ induced by AGEs were significantly larger in DM than in control group, which could be suppressed by inhibition of RAGE and ERK. However, all the cellular events of SMC_V_ were not found in paired SMC_A_. This study suggests that AGEs‐RAGE could induce the proliferation of SMC_V_ but not SMC_A_ *via* MAP kinase pathway in DM. It is the intrinsic ‘inactive’ tendency of SMC_A_ that contributes to the different rates of graft disease between SV and ITA after CABG.

## Introduction

Saphenous vein (SV) is the most commonly employed grafts in routine coronary artery bypass graft (CABG) procedure 1, 2. Unfortunately, according to the literature, within 10 years, vein graft failure occurs in nearly half of the conduits 3. Additionally, patients with diabetic mellitus (DM) tend to have accompanying more advanced, rapidly progressing vein graft disease compared with non‐diabetic patients 4, 5, 6. The leading cause of vein graft failure is intimal hyperplasia, which is defined as excessive smooth muscle cells (SMC) proliferation and migration in the intima of SV graft wall 7, 8, 9. It is facilitated by the secretion of growth factors, cytokines and matrix metalloproteinases (MMP) from endothelia and SMC themselves through the activation of key signalling pathways, including mitogen activated protein kinase (MAPK) pathway 10, 11, 12. Recent studies showed that accumulation of advanced glycation end products (AGEs) and activation of receptor of AGEs (RAGE) were found to accelerate the vascular remodelling in diabetic patients 13, 14, 15. Therefore, in this study, we chose to investigate the proliferation and MMP‐2, MMP‐9 expression of SMC from SV induced by AGEs‐RAGE *via* MAPK signalling pathway in diabetic patients.

On the other hand, internal thoracic arteries (ITA) are another commonly used graft in coronary surgical practice. As well known, ITA grafts show the overwhelming superiority to SV grafts due to their long‐term patency rates 16. However, until now, the mechanism for the inherent function differences between venous and arterial SMC has not been fully elucidated. Thus, in the present study, we also explored whether AGEs‐RAGE/MAPK signalling pathway could induce different reactions in paired SMC from SV (SMC_V_) and ITA (SMC_A_) under controlled conditions.

## Material and methods

### Study participants

The study was approved by the local Research Ethics Committee, and each patient gave written informed consent. Twenty‐five individuals with type 2 DM had fasting blood glucose >5.5 mmol/l and/or current treatment with insulin or oral hypoglycaemic agents. The non‐diabetic (control) group comprised the other 25 patients who had no pre‐operative DM history. Patients previously diagnosed as hyperlipidaemia (total cholesterol level >200 mg/dl) or hypertensive (systolic blood pressure >140 mmHg, diastolic blood pressure >90 mmHg) were on appropriate lipid‐lowering or hypotensive agent treatments. Individual characteristics of the patients are presented in Table 1.

**Table 1:** The Demographics of the Studied Population

	Control (*n* = 25)	DM group (*n* = 25)	*P*‐value
Gender (male/%)	20 (80.0)	22 (88.0)	0.18
Age (year)	62.8 ± 10.5	63.2 ± 11.8	0.53
Bodyweight (kg)	66.3 ± 14.7	64.7 ± 11.3	0.46
Smoking (*n*/%)	11 (44.0)	9 (36.0)	0.07
Hypertension (*n*/%)	12 (48.0)	14 (56.0)	0.09
Hyperlipidaemia (*n*/%)	8 (32.0)	9 (36.0)	0.11
Cerebral vessel disease (*n*/%)	9 (36.0)	10 (40.0)	0.14
Periphery vessel disease (*n*/%)	6 (24.0)	7 (28.0)	0.10
Chronic renal failure (*n*/%)	3 (12.0)	4 (16.0)	0.08
Chronic obstructive pulmonary disease (*n*/%)	4 (16.0)	3 (12.0)	0.08
Diabetic history (year)	…	10.5 ± 4.7	…
Fasting blood glucose (mmol/l)	5.2 ± 1.1	14.17 ± 5.63	<0.01

### SMC culture

Samples of SV and ITA were obtained at the time of CABG operation. Parts of the grafts were minced and cultured in DMEM contained 10% foetal calf serum (FCS) until SMC came out. The cells were then maintained in DMEM containing 10% FCS in a humidified 5% CO_2_/95% air atmosphere at 37°C.

### Preparation of AGEs

Advanced glycation end products were prepared as previously reported 17. Briefly, bovine serum albumin (BSA) was incubated with 0.5 mol/l glucose in PBS in the dark for 16 weeks at 37°C in the presence of 1.5 mmol/l phenylmethylsulphonyl fluoride, 0.5 mmol/l ethylenediaminetetraacetic acid, penicillin (100 U/ml) and streptomycin (100 U/ml) under sterile conditions. Control non‐glycated BSA was incubated in the absence of glucose under the same conditions. The concentration of the AGEs‐BSA solution used in this study was 100 μg/ml. The AGEs‐BSA solution was confirmed to be endotoxin free (<0.5 U/ml of endotoxin).

### SMC proliferation assays

To assess the different pre‐existing proliferative property, paired SMC_V_ and SMC_A_ were seeded in parallel into 24‐well tissue culture plates at a density of 1 × 10^4^ cells per well in full growth medium (DMEM plus 10% FCS). The medium was replaced every 2 days. After 5 days, the cells were released from culture wells and Cell viability was monitored by MTT (3‐(4,5‐dimethylthiazol‐2‐yl)‐2,5‐ diphenyltetrazolium bromide) assay. Cell were seeded onto 96‐well plates (1 × 10^4^ cells/well). After treatment, MTT tetrazilium salt (Sigma‐Aldrich, St. Louis, MO, USA., 0.2 mg/ml) was added to each well, cells were further incubated in 5% CO_2_ at 37°C for 4 hrs. DMSO was added to dissolve formazan crystals for 20 min. The number of viable cells was assessed by measurement of the absorbance at 490 nm using a Safire 2 microplate reader (TECAN, San Jose, CA, USA).

To study the effect of AGEs on SMC proliferation, cells were seeded at 1 × 10^4^ cells per well. After 24 hrs, the full growth medium was changed, and the cells were incubated with fresh medium containing AGEs‐BSA (100 μg/ml) for 12 hrs or pre‐treated with anti‐RAGE antibodies (20 μg/ml) for 1 hr and then incubated with AGEs‐BSA (100 μg/ml) for 12 hrs. and then full growth medium was changed, the cells were continued to culture for 24 hrs.

### Real‐time PCR

Real‐time PCR was performed with an Applied Biosystems 7300 Real‐time PCR System with TaqMan Universal PCR Master Mix and TaqMan Gene Expression Assays (Applied Biosystems, Foster City, CA, USA). The amplification cycle consisted of 2 min. at 50°C, 10 min. at 95°C, 15 sec. at 95°C, and 1 min. at 60°C. GAPDH served as controls for PCR. Relative gene expression levels were quantified using the 2^(−∆∆Ct)^ formula.

### Western Blot analysis

Total proteins isolated from vessels or culture SMC were size‐fractionated by SDS–PAGE and transferred to Immobilon‐P membranes (Millipore, Billerica, MA, USA). Each membrane was incubated with specific primary antibodies (Calbiochem, San Diego, CA, USA) and subsequently with secondary antibody (Jackson Immunolab, West Grove, PA, USA). GAPDH or β‐actin served as the internal controls. Immune complexes were visualized with the enhanced chemiluminescence detection system (Amersham, Piscataway, NJ, USA). Quantification of bands was performed by densitometry using the LAS‐3000 Imaging System (FUJIFILM, Kanagawa, Japan) and NIH Image J software.

### Immunofluorescent stain analysis

Serial 3‐μm paraffin sections of paired ITA and SV were dewaxed and rehydrated. After the sections were blocked, sections were incubated for 1 hr at room temperature with primary antibodies (Santa Cruz Biotechnology Inc., Dallas, TX, USA) diluted in 1.5% BSA in PBS. Paired SMC_A_ and SMC_V_ were washed by PBS, fixed with 4% paraformaldehyde and permeabilized with 0.1% Triton X‐100. After the cells were incubated with BSA for 30 min., the primary antibodies were added overnight at 4°C. The sections and SMC were rinsed, incubated with Fluorescein isothiocyanat (FITC)‐conjugated (Invitrogen, Carlsbad, CA, USA.) or Cy3‐conjugated (Jackson ImmunoResearch) secondary antibodies for 1 hr at room temperature in the dark room, and then counterstained with 4′,6′‐diamidino‐2‐phenylindole (DAPI; Sigma‐Aldrich). The fluorescent signals were observed under the fluorescence microscope (Zeiss, Munich, Germany).

### Statistical analysis

All values were expressed as means ± S.D. Paired and/or unpaired Student's *t*‐tests were used as appropriate to evaluate the statistical significance of differences between two group means, and anova was performed for multiple groups by one‐way anova. *P* < 0.05 was considered statistically significant. Statistical analysis was performed with SAS 6.12 software (SAS Institute Inc, Chicago, IL, USA).

## Results

### Demographic characteristics of patients

The demographics of the studied population did not differ in the two groups except for parameters related to diabetic history. The two groups were well‐matched for age, gender and presentation profile (Table 1).

### AGEs has different effects on proliferation of SMC_V_ and SMC_A_

**Figure 1:**
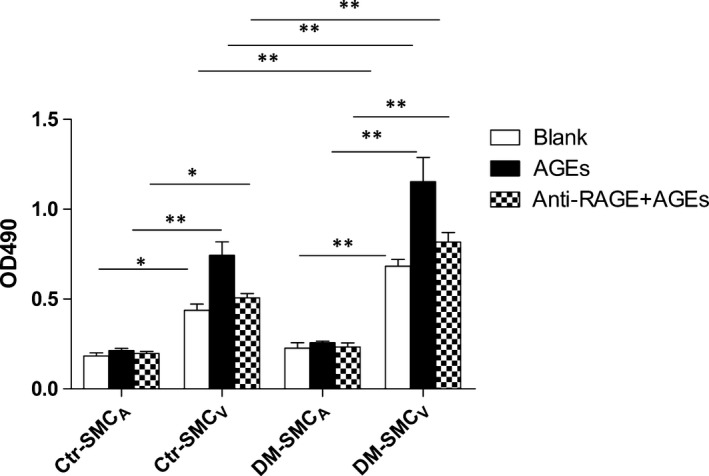
AGE‐induced proliferation of paired SMC_A_ and SMC_V_. Bar graphs show the endogenous proliferative properties of paired SMC_A_ and SMC_V_ obtained from patients with (DM) or without (control) diabetes. Ctr means control group. DM means diabetic group. AGEs means SMC incubated with fresh medium containing AGEs‐BSA (100 μg/ml) for 12 hrs anti‐RAGE+AGEs means SMC neutralized with anti‐RAGE antibodies (20 μg/ml) for 1 hr and then incubated with AGEs‐BSA (100 μg/ml) for 12 hrs. Data are shown as means ± S.D. (*N* = 8). *P < 0.05; **P < 0.01.

Compared with paired SMC_A_, SMC_V_ showed increased proliferative property in both of the two groups (Fig. 1, blank). And the difference of the inherent proliferative property between paired SMC_V_ and SCM_A_ was more noticeable in DM group than the control group (*P* < 0.05 and <0.01 in control and DM group, respectively), indicating that SMC_V_ in DM group showed more significantly increased proliferation than SMC_V_ in control group. Interestingly, there was no difference of proliferation in SMC_A_ between the two groups (*P* > 0.05). In the stimulatory study, it was observed that the SMC_V_ treated with AGEs‐BSA showed significantly increased proliferation than paired SMC_A_ in both groups (Fig. 1, AGEs, *P* < 0.01), and the increase in DM group was significantly larger than in control group (*P* < 0.01). However, the proliferative increase of SMC_A_ induced by AGEs‐BSA in the two groups did not reach statistical significance (*P* > 0.05). Furthermore, pre‐treating cells with RAGE‐neutralizing antibody could attenuate AGEs‐induced proliferative increases of SMC_V_ in both groups. These results indicate that AGEs‐RAGE can induce the increase in the proliferation of SMC_V_ but not SMC_A_, and the increase is more significant in patients with DM than those without DM.

### Transcript expression levels of MAPK family and MMPs mRNA in SMC induced by AGEs

**Figure 2:**
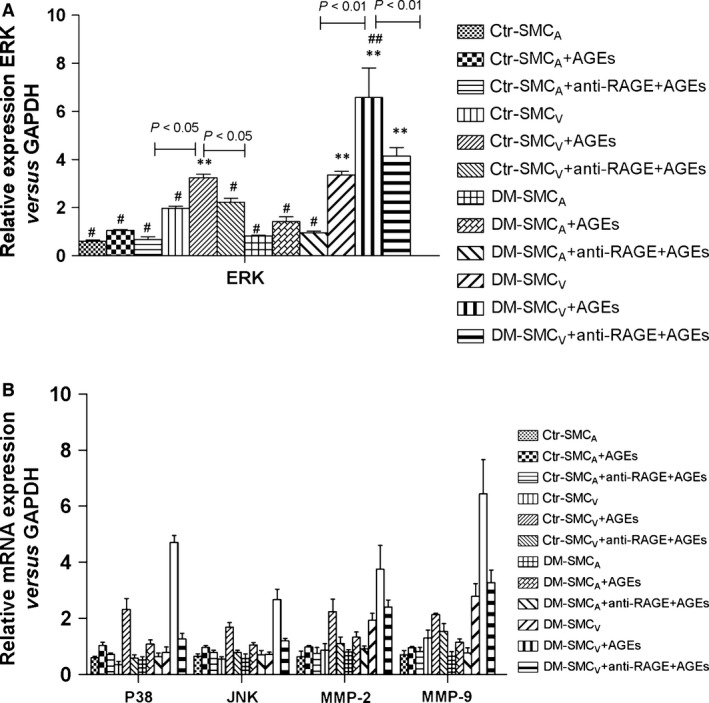
Transcript expression levels of MAPK family and MMPs mRNA in paired SMC induced by AGEs. The transcript expression levels of MAPK family (ERK, p38, and JNK), MMP‐2 and MMP‐9 mRNA were analysed by real‐time PCR in paired SMC_A_ and SMC_V_ obtained from patients with (DM) or without (control, Ctr) DM. (**A**) ERK. (**B**) p38, JNK, MMP‐2 and MMP‐9. +AGEs means SMC incubated with fresh medium containing AGEs‐BSA (100 μg/ml) for 12 hrs +anti‐RAGE+AGEs means SMC neutralized with anti‐RAGE antibodies (20 μg/ml) for 1 hr and then incubated with AGEs‐BSA (100 μg/ml) for 12 hrs. The transcript expression levels were calculated as the ratio to GAPDH expression. Data are shown as means ± S.D. (*N* = 8). ** *versus* #, *P* < 0.05; ** *versus* ##, *P* < 0.01; ## *versus* #, *P* < 0.01.

Mitogen activated protein kinase pathway is important for cells to respond to numerous growth factors. Matrix metalloproteinases have the effects on promoting the migration of SMC. We therefore examined the gene expression of these molecules (Fig. 2). Compared with paired SMC_A_, SMC_V_ showed increased transcript expression of MAPK family (ERK, p38, and JNK), MMP‐2 and MMP‐9 mRNA in both of the two groups, and the difference of the expression between paired SMC_V_ and SCM_A_ was more noticeable in DM group than the control group (Fig. 2, *P* < 0.05 and <0.01 in control and DM group, respectively), indicating that SMC_V_ in DM group showed more significantly increased proliferation than SMC_V_ in control group. However, there was no difference of the expression in SMC_A_ between the two groups (*P* > 0.05). After stimulation with AGEs, transcript expression levels of the MAPK family and MMP‐2/9 gene were further increased in SMC_V_ of the two groups (Fig. 2, SMC_V_ plus AGEs *versus* that without AGEs, *P* < 0.05 and *P* < 0.01 in control and DM, respectively). And this phenomenon was more notable in DM group (*P* < 0.01 *versus* control group). In contrast with these changes in SMC_V_, the relative transcript expressions of these genes had no significant difference in SMC_A_ from both groups (*P* > 0.05). RAGE antibody treatment could reduce transcript expression levels of MAPK family and MMPs mRNA in SMC_V_ compared to that of the untreated SMC_V_, *P* < 0.05, respectively. However, it was not found in SMC_A_ of the two groups, *P* > 0.05, respectively. These data suggest that AGEs‐RAGE can elevate the transcript expression levels of MAPK family and MMPs mRNA in SMC_V_, but not in SMC_A_, especially in DM group.

### Phosphorylation of MAPK family and expression of MMPs proteins in SMC

**Figure 3:**
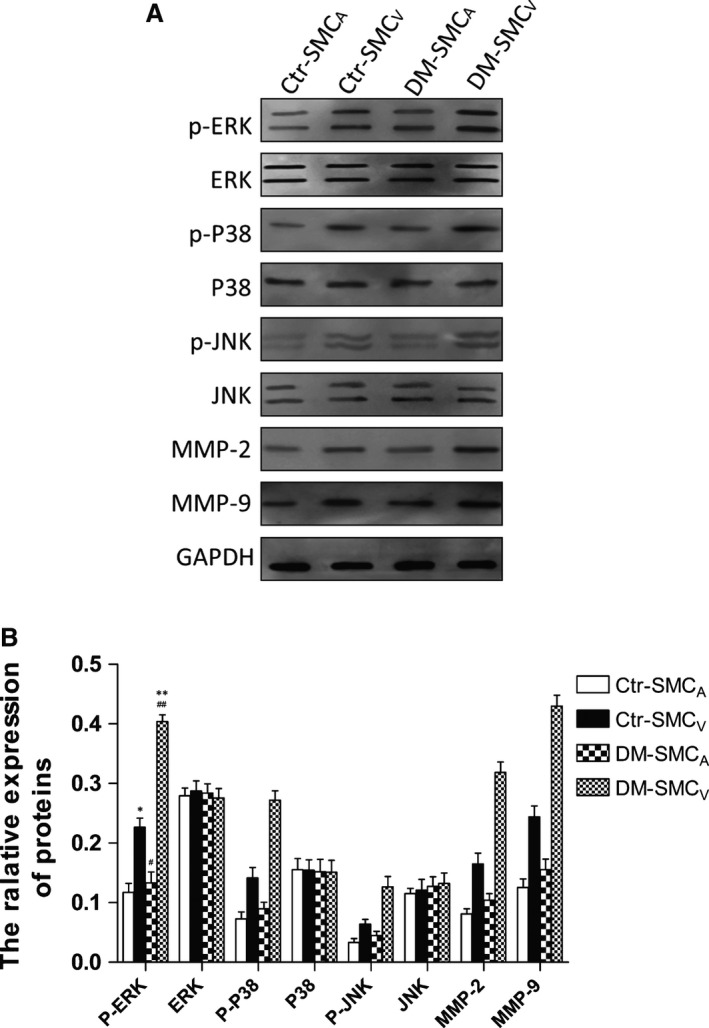
The native protein levels of MAPK family, MMP‐2 and MMP‐9 in paired SMC. The native protein phosphorylation or expression levels of MAPK family, MMP‐2 and MMP‐9 were analysed by Western blotting in paired SMC_A_ and SMC_V_ obtained from patients with (DM) or without (control, Ctr) DM. (**A**) Representative images showing phosphorylated (p‐) and total protein levels of MAPK family (ERK, p38, and JNK), MMP‐2 and MMP‐9 in paired SMC_A_ and SMC_V_. GAPDH served as an internal control. (**B**) Quantitative analysis of p‐ERK, ERK, p‐p38, p38; p‐JNK, JNK, MMP‐2 and MMP‐9 expressions. The expression level was calculated as the ratio to GAPDH expression. Data are shown as means ± S.D. (*N* = 8). *P < 0.05, compared with SMC_A_; ** *versus* *, *P* < 0.05; ## *versus* #, *P* < 0.01.

**Figure 4:**
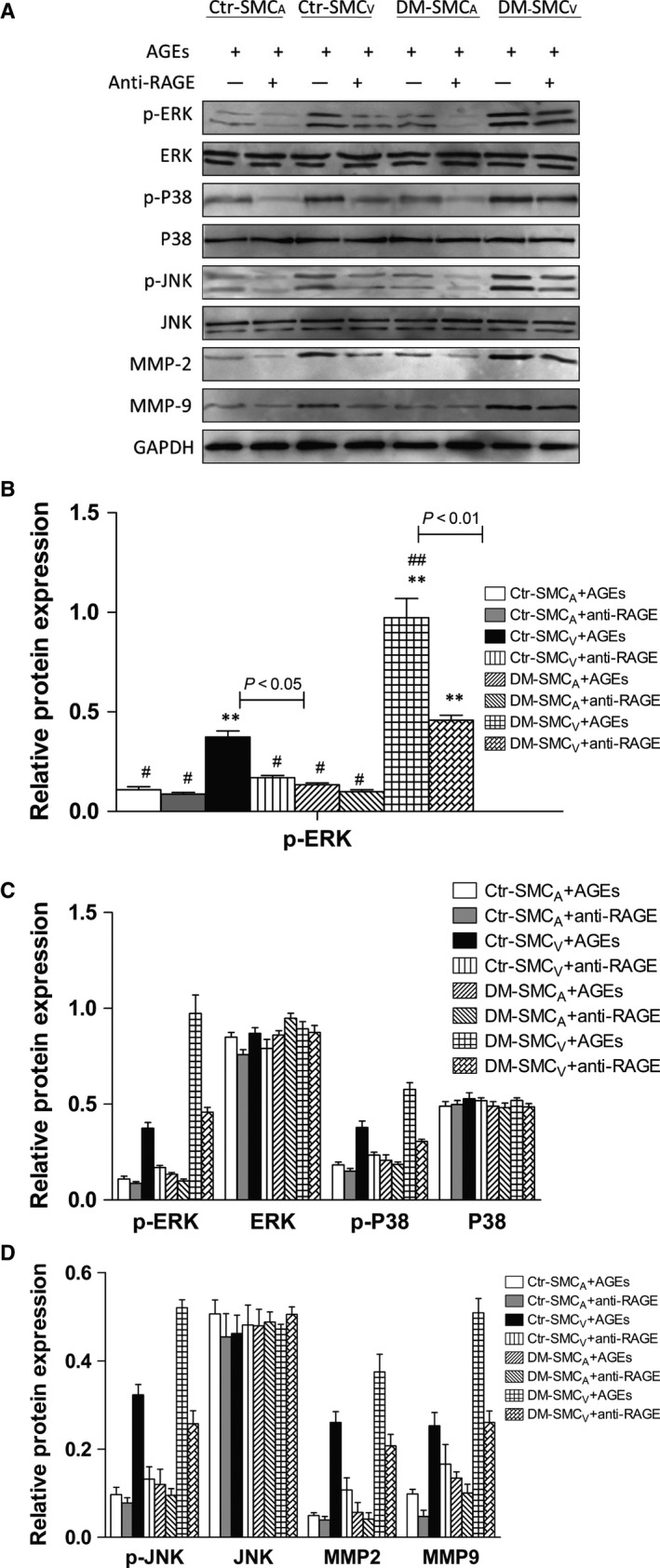
The effect of AGEs on the protein levels of MAPK family and MMP‐2, MMP‐9 in paired SMC. The protein phosphorylation or expression levels of MAPK family and MMPs were analysed by Western blotting in paired SMC_A_ and SMC_V_ obtained from patients with (DM) or without (control, Ctr) DM. (**A**) Representative images are shown. GAPDH served as an internal control. (**B** and **C**) Statistical analysis for the expression levels of p‐ERK, ERK, p‐p38, p38, p‐JNK, JNK, MMP‐2 and MMP‐9, respectively. Values are shown as the radio to GAPDH expression. AGEs+ means SMC incubated with fresh medium containing AGEs‐BSA (100 μg/ml) for 12 hrs anti‐RAGE+ means SMC neutralized with anti‐RAGE antibodies (20 μg/ml) for 1 hr and then incubated with AGEs‐BSA (100 μg/ml) for 12 hrs. Data are expressed as means ± S.D. (*N* = 8). ** *versus* #, *P* < 0.05; ** *versus* ##, *P* < 0.01; ## *versus* #, *P* < 0.01.

To further investigate the different reactions of paired SMC_V_ and SMC_A_ induced by AGEs, we examined the phosphorylation levels of various MAPK family proteins and expression levels of MMP‐2/9 proteins in SMC by Western blotting. Compared with paired SMC_A_, SMC_V_ showed increased phosphorylation levels of ERK, p‐38 and JNK and protein expression levels of MMP‐2/9 in both of the two groups (Fig. 3A and B, p‐ERK, p‐p38, p‐JNK). And the difference in the phosphorylation and the protein expression between paired SMC_V_ and SCM_A_ was bigger in DM group than the control group (Fig. 3B, *P* < 0.05 and *P* < 0.01 in control and DM, respectively; DM *versus* control, *P* < 0.05). Interestingly, there was no difference in the phosphorylation or expression of these proteins in SMC_A_ between the two groups (*P* > 0.05). Moreover, after stimulation with AGEs, compared with SMC_V_ without the stimulation, the levels of p‐ERK, p‐p38, p‐JNK, MMP‐2 and MMP‐9 proteins were significantly more increased in SMC_V_ but not in SMC_A_ from the both groups (Fig. 4A–D, +AGEs, *P* < 0.05 and *P* < 0.01 in control and DM, respectively), and this phenomenon was more notable in DM group (*P* < 0.01 *versus* control group). RAGE antibody treatment could reduce all the changes in the phosphorylation of MAPK family and the expression of MMPs proteins in SMC_V_ compared to that of the untreated SMC_V_, *P* < 0.05, respectively (Figs 3 and 4). These data suggest that AGEs‐RAGE can promote the phosphorylation of MAPK family and the expression of MMPs protein in SMC_V_ but not in SMC_A_, especially in DM group.

### MAPK family is involved in AGEs‐induced expression of MMPs in SMC_V_

**Figure 5:**
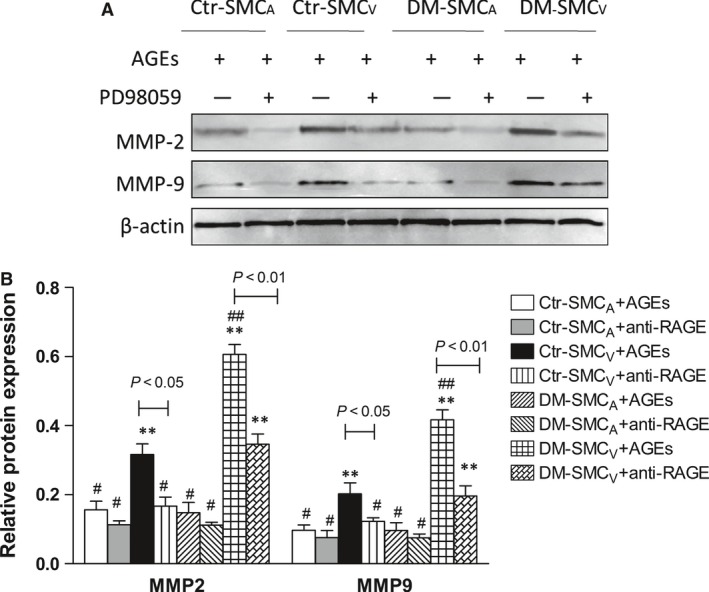
The role of ERKs in AGE‐induced expression of MMP‐2 and MMP‐9 in paired SMC. Paired SMC obtained from patients with (DM) or without (control, Ctr) DM were pre‐treated with the ERK inhibitor PD98059 (20 μM) (+) or vehicle (‐) for 30 min. and then incubated with AGEs‐BSA (100 μg/ml) for 12 hrs. The expression of MMP‐2 and MMP‐9 protein was analysed by Western blotting. (**A**) Representative images are shown. β‐actin served as an internal control. (**B**) Quantitative analysis of MMP‐2 and MMP‐9 expressions. Values represent the radio to β‐actin expression. Data are expressed as means ± S.D. (*N* = 8). ** *versus* #, *P* < 0.05; ** *versus* ##, *P* < 0.01; ## *versus* #, *P* < 0.01.

To ask whether AGEs‐RAGE induces the expression of MMPs in SMCv through MAPK family, we used an inhibitor of ERK, PD98059 to pre‐treat the cells. Pre‐treating with PD98059 significantly abrogated AGEs‐induced expression of MMP‐2 and MMP‐9 in SMC_V_ from both of the two groups (Fig. 5A and B, *P* < 0.05 or *P* < 0.01, respectively). The data suggest that AGEs stimulates the expression of MMP‐2 and MMP‐9 proteins through MAPK family in SMC_V_, but not in SMC_A_.

### Coexpression of RAGE and MMPs in SMC

**Figure 6:**
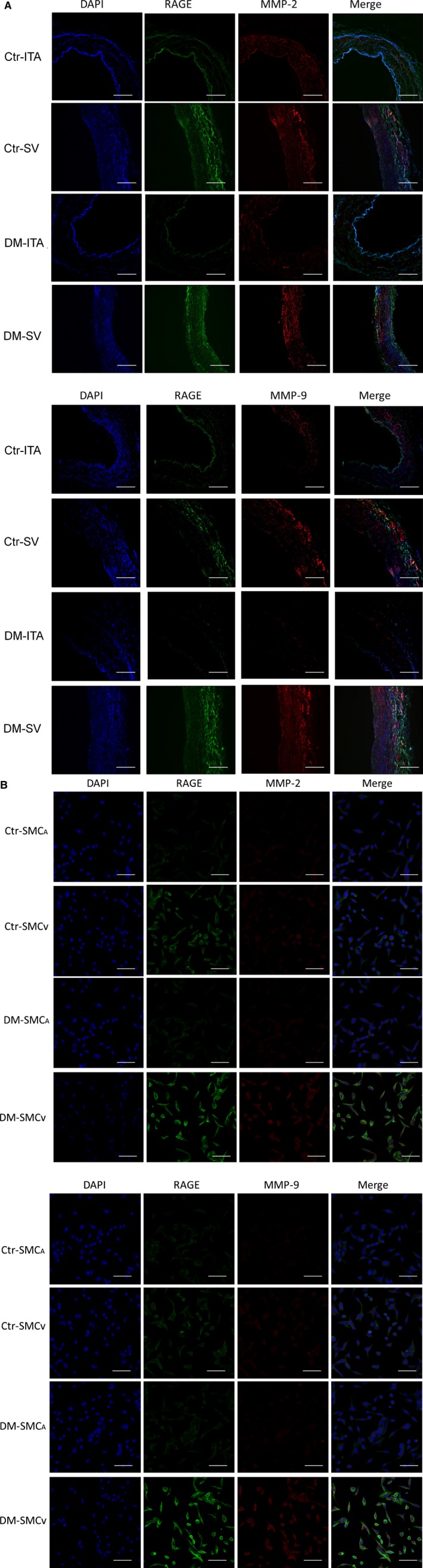
Coexpression of RAGE and MMPs in paired ITA and SV and paired SMC_A_ and SMC_V_. Paired ITA and SV and paired SMC_A_ and SMC_V_ obtained from patients with (DM) or without (control, Ctr) DM were doubly labelled with anti RAGE and MMP‐2 or anti RAGE and MMP‐9 antibodies, respectively, by immunofluorescent staining. Respective two sets of representative photos of paired ITA and SV and paired SMC_A_ and SMC_V_ from eight experiments are shown. Green and red fluorescence indicate RAGE and MMP‐2/9 signals, respectively. Cell nuclear are stained by DAPI. (**A**) Paired ITA and SV. (**B**) Paired SMC_A_ and SMC_V_. Original magnification ×100.

To further confirm the above findings *in vitro*, we finally tested the coexpression of RAGE and MMPs in paired SV and ITA *in vivo* and SMC_V_ and SMC_A_ *in vitro* by immunofluorescent staining. The results revealed that compared with the paired ITA or SMC_A_, the fluorescent signals labelled with RAGE (FITC), MMP‐2 and ‐9 (Cy3) were significantly increased in the media of SV or SMC_V_ in both groups, especially in DM group (Fig. 6A and B). However, the fluorescent signals of these proteins had no significant difference in the media of ITA or SMC_A_ in both groups (Fig. 6A and B). Double labelled immunofluorescent results distinctly confirmed that the relations between protein levels of RAGE and MMPs had significant difference in the media between paired SV and ITA conduits or paired SMC_V_ and SMC_A_.

## Discussion

Saphenous vein is the most commonly used conduit in CABG procedure, although it is prone to post‐operative graft disease 1, 2. Many studies have attempted to elucidate the mechanism of vein graft disease 18, 19, 20. Intima hyperplasia serves as the foundation for subsequent progressive graft atheroma which eventually results in occlusion of the SV grafts years after CABG 21, 22. Abnormal proliferation and migration of SMC from the media to the intima through internal elastic lamina are key events in the development of intima hyperplasia 23. Before migration of SMC, the surrounding extracellular matrix must be initially degraded by the matrix metalloproteinases MMPs 24. Of several MMPs, MMP‐2, MMP‐9 possess the unique ability to degrade elastin and collagen, the main components of the basilar membrane 25. However, the relation between MMPs in SMC and SV graft disease remained unknown. We here clearly demonstrate precise molecular and cellular mechanisms involved in the native pathological remodelling of SV in diabetic patients characterized by SMC proliferation and MMPs over‐expression.

The metabolic effects of hyperglycaemia in diabetic patients may render them vulnerable to vascular complications 3, 26. However, even when blood glucose is well controlled, intimal hyperplasia is more prevalent in vein grafts of diabetic patients, suggesting that additional factors contribute to their poor outcome 27. Some studies suggest that AGEs accelerate atherosclerosis in diabetic patients with coronary heart disease 13, 14, 15. Advanced glycation end product‐induced cell autophagy has been shown to contribute to the process of proliferation of Vascular Smooth Muscle Cells (VSMCs), which is related to atherosclerosis in diabetes 15. Advanced glycation end products may also induce calcification of VSMCs by osteoblast‐like differentiation of the cells through RAGE/p38 MAPK signalling pathway 28. Moreover, proliferation of SMC, a key factor in the development of atherosclerotic lesions, is significantly stimulated by the accumulation of AGEs and their interaction with RAGE 29. Activation of RAGE not only accelerates early lesion formation but sustains lesion progression in the diabetic *apoE*‐null mouse model 30. RAGE activation in VSMC could induce the development of vascular diseases by interfering with the contractile phenotype of VSMC through the modification of their mechanical and functional properties 31. In this study, we found that the interaction between AGEs and RAGE significantly increased proliferation and secretion of MMP‐2/9 of SMC_V_ *via* MAPK signalling pathway. SMC_V_ from diabetic patients showed intrinsically much higher endogenous proliferation and secretion of MMPs than those from non‐diabetic individuals. The increased SMC_V_ proliferation in diabetic patients was associated with AGEs‐RAGE interaction and activation of MAPK signalling pathway might be one of the pathophysiological mechanisms. Additionally, this may also provide reasonable evidence for why there are so more stenosis and occlusion of SV grafts in diabetic patients.

On the other hand, numerous studies have shown the superiority of ITA in promoting long‐term survival and reduced recurrence of major adverse cardiac events after CABG in all patients, including those with DM 16, 32. Some studies about why graft disease is more prevalent in SV than ITA have focused on the effects from surgical trauma during operative manipulation and altered hemodynamic conditions 33, 34. However, theory is growing that SMC_V_ and SMC_A_ are derived from different embryonic origins and exhibit distinct functions which may contribute to the different rate of graft disease observed in SV *versus* ITA grafts 34. This raises the question of whether SMC_V_ is intrinsically more ‘active’ than paired SMC_A_.

Some studies have revealed that AGEs have been shown to induce proliferation SMC, increase generation of reactive oxygen species, decrease nitric oxide bioavailability and up‐regulate the production of various cytokines or growth factors, such as tumour necrosis factor‐α, platelet‐derived growth factor and Vascular Cell Adhesion Molecule 1 (VCAM‐1) 35, 36. Human SV‐SMCs are inherently more proliferative and invasive than paired IMA‐SMCs, likely due to a relative increase in p44/42‐MAPK activation 23. However, even now, there is no direct evidence for the reaction in SMC_A_ induced by AGEs‐RAGE through MAPK signalling pathway in diabetic conditions. Therefore, we explored whether AGEs‐RAGE/MAPK signalling pathway could induce different reactions in paired SMC_V_ and SMC_A_ under controlled conditions. In this study, the activation of SMC_V_ induced by AGEs‐RAGE/MAPK signalling pathway was not found in SMC_A_ from any subgroups in our study. SMC_A_ exhibited much lower inherent proliferation, MAKP family protein phosphorylation and MMPs secretion compared with paired SMC_V_ in both diabetic and non‐diabetic subgroups. It is the intrinsic ‘inactive’ tendency of SMC_A_ that may contribute to the obviously different rates of graft disease between SV and ITA after coronary surgery.

PI3K/Akt signalling pathway, particular the p‐Akt is required to test for elucidating the mechanism of SMC proliferation. We also examined the expression of PI3K/p‐PI3K and Akt/p‐Akt. The results show that there are no significant differences in the activation of PI3K/Akt after stimulation with AGE between paired SMC_A_ and SMC_V_ (Fig. S1), suggesting that PI3K/Akt signalling is not involved in the mechanisms underlying the different graft failure rate between SV and ITA grafts. The precise reason for why MAPK family, but not Akt, contributes to AGEs‐induced MMPs secretion and SMC_V_ proliferation in DM remains to further study.

Nevertheless, these observations raise the question of whether a local genetic treatment should be considered in diabetic patients with venous bypass grafts. Novel therapeutic approaches may develop that transform the SV conduit into a vessel with an increased long‐term patency. We hope our study may provide novel information for further understanding graft failure in diabetic patients after CABG procedure.

## Conflicts of interest

The authors confirm that there are no conflicts of interest.

## Supporting information


**Figure S1** The effect of AGEs on the phosphorylation and expression levels of PI3K and AKT proteins in paired SMC.
