# Supplementary material for: Advanced glycation end products impair the functions of saphenous vein but not thoracic artery smooth muscle cells through RAGE/MAPK signalling pathway in diabetes
Source: J Cell Mol Med. 2016 Jun 14;20(10):1945–55. doi: 10.1111/jcmm.12886 (PMC5020631; doi:10.1111/jcmm.12886)
Supplement: Supplementary file 1 — Figure S1 The effect of AGEs on the phosphorylation and expression levels of PI3K and AKT proteins in paired SMC. [file JCMM-20-1945-s001.docx]

**Supplemental Figure**

**A**


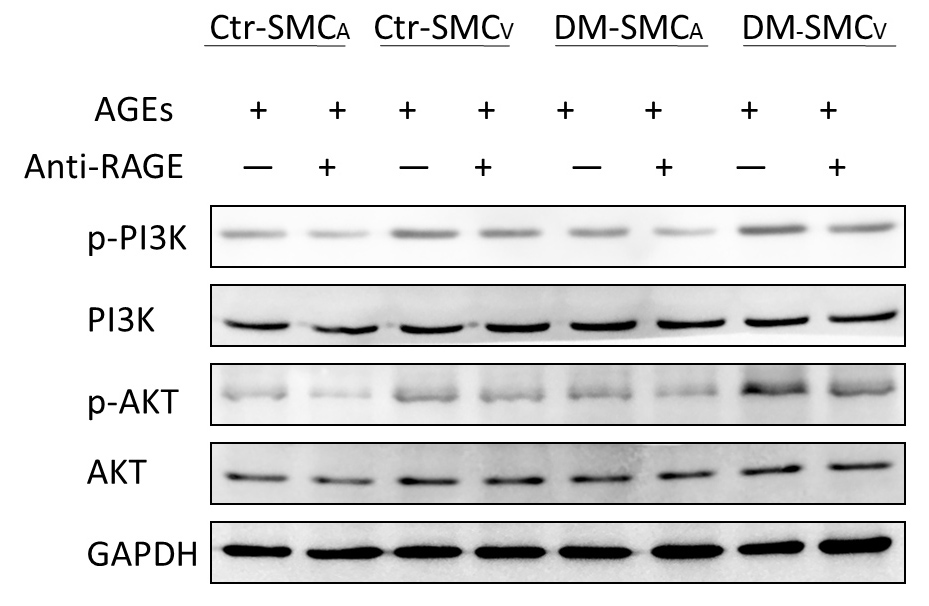


**B**


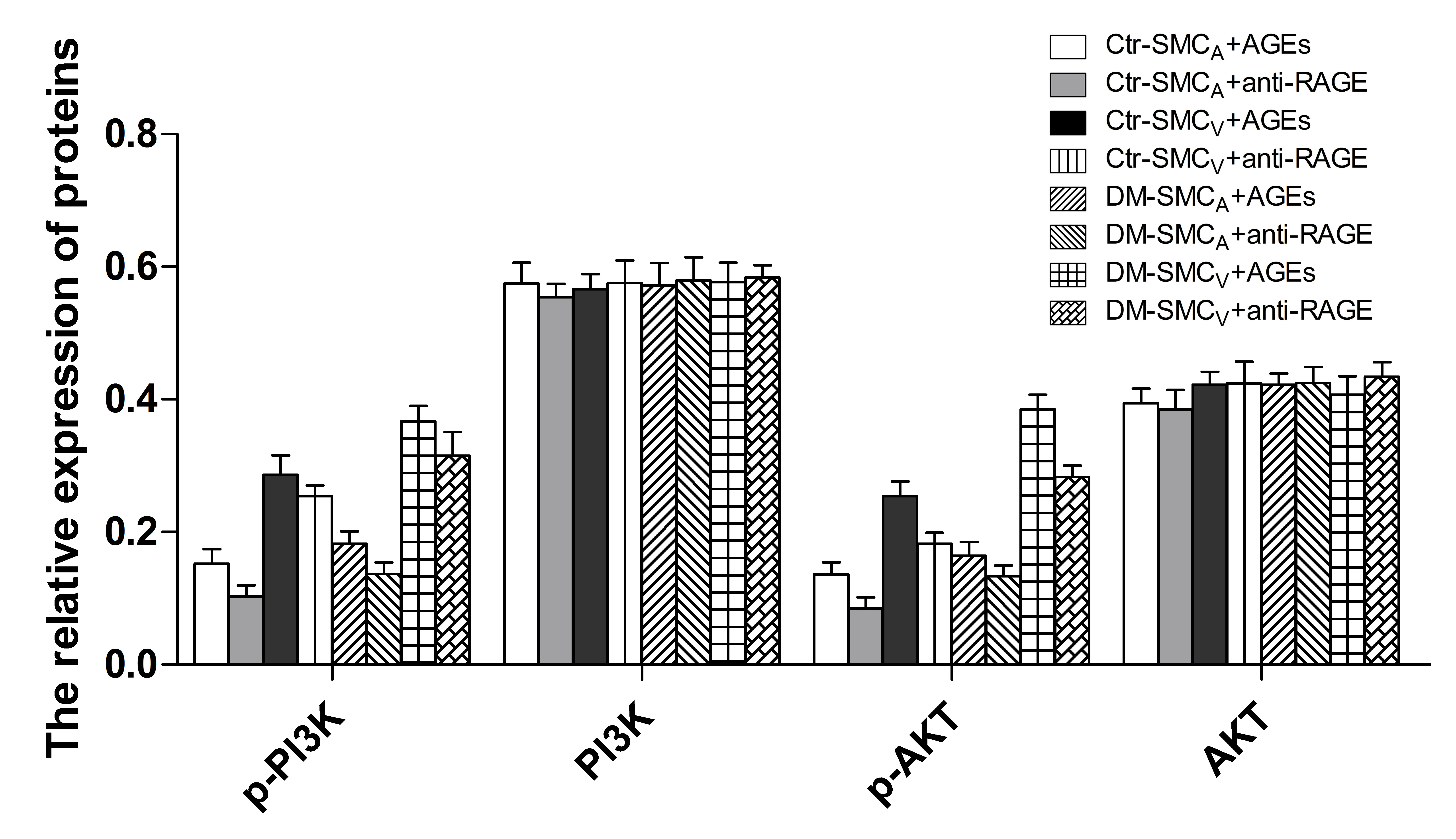


**Supplemental Figure Legend**

**The effect of AGEs on the phosphorylation and expression levels of PI3K and AKT proteins in paired SMC**

The protein phosphorylation and expression levels of PI3K and AKT were analyzed by Western blotting in paired SMC_A_ and SMC_V_ obtained from patients with (DM) or without (control, Ctr) DM. **A**, Representative images are shown. GAPDH served as an internal control. **B**, Statistical analysis for the phosphorylation and expression of PI3K and AKT, respectively. Values are shown as the radio to GAPDH expression. AGEs+ means SMC incubated with fresh medium containing AGEs-BSA (100 μg/mL) for 12 h. anti-RAGE+ means SMC neutralized with anti-RAGE antibodies (20 µg/mL) for 1 h and then incubated with AGEs-BSA (100 µg/mL) for 12 h. Data are expressed as means ± SD (N=8). There were no signifcant differences between SMC_V_ treated with AGEs and with anti-RAGE+ AGEs in both the control and DM groups (*p* > 0.05)
